# Loss of Fshr Prevents Testicular Maturation in Atlantic Salmon (*Salmo salar* L.)

**DOI:** 10.1210/endocr/bqae013

**Published:** 2024-01-31

**Authors:** Eva Andersson, Rüdiger W Schulz, Fernanda Almeida, Lene Kleppe, Kai Ove Skaftnesmo, Erik Kjærner-Semb, Diego Crespo, Per Gunnar Fjelldal, Tom Johnny Hansen, Birgitta Norberg, Rolf B Edvardsen, Anna Wargelius

**Affiliations:** Institute of Marine Research, NO-5817 Bergen, Norway; Institute of Marine Research, NO-5817 Bergen, Norway; Science Faculty, Department Biology, Utrecht University, NL-3584 CH Utrecht, The Netherlands; Embrapa Amazônia Ocidental, 69010-97 Manaus, Amazonas, Brazil; Institute of Marine Research, NO-5817 Bergen, Norway; Institute of Marine Research, NO-5817 Bergen, Norway; Institute of Marine Research, NO-5817 Bergen, Norway; Institute of Marine Research, NO-5817 Bergen, Norway; Institute of Marine Research, NO-5817 Bergen, Norway; Institute of Marine Research, NO-5817 Bergen, Norway; Institute of Marine Research, NO-5817 Bergen, Norway; Institute of Marine Research, NO-5817 Bergen, Norway; Institute of Marine Research, NO-5817 Bergen, Norway

**Keywords:** FSH receptor, CRISPR-Cas9, puberty, sterility, aquaculture

## Abstract

Early puberty poses a significant challenge for male Atlantic salmon in aquaculture due to its negative impact on growth and welfare. The regulation of puberty in vertebrates involves 2 key reproductive hormones: follicle-stimulating hormone (FSH) and luteinizing hormone (LH) and their gonadal receptors. In male mice lacking FSH receptor, testes size is reduced, but fertility is maintained, while medaka and zebrafish with a disrupted *fshr* gene exhibit near normal testis size and fertility. In these fishes both Fsh and Lh are present during puberty and Lh may rescue fertility, while in salmonid fish only Fsh is present in the circulation during puberty. Using CRISPR-Cas9, we produced crispants with a high prevalence of *fshr* mutations at the target site, which remained fertile, although more than half showed a testis development deviating from wild-type (wt) males. Crossing out these F0 crispants to each other produced a viable F1 generation showing frameshift (*fshr^−/−^*) or in-frame mutations (*fshr^if/if^*). Nearly all wt males matured while all *fshr^−/−^* males remained immature with small testes containing A spermatogonia as the furthest developed germ cell type and prepubertal plasma androgen levels. Also, the pituitary transcript levels of *gnrhr2bba* and *lhb*, but not for *fshb*, were reduced in the *fshr^−/−^* males compared with maturing males. More than half of the *fshr^if/if^* mutant males showed no or a delayed maturation. In conclusion, Atlantic salmon show the unique characteristic that loss of Fshr function alone results in male infertility, offering new opportunities to control precocious puberty or fertility in salmon.

Early puberty is a major problem in farmed Atlantic salmon males. Growth is stunted and the elevated sex steroid plasma levels characteristic of puberty trigger welfare problems, such as an increased disease susceptibility and the loss of osmoregulatory capacity in seawater (1). High incidences of puberty are also observed in Atlantic salmon postsmolts cultured in land-based water recirculation aquaculture systems (RASs) (2) that are increasingly used to avoid infections with a parasitic crustacean, the salmon lice. A better understanding of the regulation of puberty will aid in the developing of approaches which increase sustainability of salmon aquaculture.

The endocrine system regulating puberty in vertebrates is the brain–pituitary–gonad axis. Neuroendocrine input to the pituitary controls the release of 2 pituitary gonadotropic hormones, follicle-stimulating hormone (FSH) and luteinizing hormone (LH), that regulate the development and adult functioning of steroidogenesis and spermatogenesis, the 2 major testicular activities (3). In mammals, the interstitial Leydig cells express the receptor for LH (LHCGR) that controls androgen production (4). Androgens have a broad spectrum of biological activities, including stimulating spermatogenesis via the androgen receptor expressed by testicular somatic cells, such as Sertoli and peritubular myoid cells (5). Functional studies in mammals showed that loss of the LHCGR, or loss of the androgen receptor from testicular somatic cells, blocked spermatogenesis (4, 5). The receptor for FSH (FSHR) is expressed by Sertoli cells (4), the only cell type within the spermatogenic tubules next to germ cells. Sertoli cells are in direct, close contact with all germ cells at all stages of their development and provide essential physical, nutritional, and regulatory support during spermatogenesis (6). Surprisingly, loss of FSHR did not lead to infertility in mice, although testis size, Sertoli cell number, quantity, and quality (eg, motility) of spermatozoa were clearly reduced in mutants (4). This led to the concept that LH/androgens, but not FSH, are strictly required for spermatogenesis. Still, a constantly active FSHR can promote nearly normal spermatogenesis even in the absence of androgen signaling in mice (7). This finding was remarkable because it was previously thought that spermatogenesis was at least in part dependent on androgen signaling. Thus, while not being strictly required for spermatogenesis, FSH alone nevertheless can enhance male fertility in mice.

Also in fish, the *lhcgr* gene is expressed by Leydig cells and Lh stimulates androgen production (8). In addition, Lhcgr protein expression occurs in postmeiotic germ cell stages and facilitates direct stimulation of spermiogenesis (9). Interestingly, loss of *lhcgr* had no detrimental effects on male fertility in zebrafish (10) or medaka (11). This is probably related to the fact that the steroidogenic Leydig cells in fish express the *fshr* and produce androgens when stimulated by Fsh (ie, the absence of Lh can be compensated by Fsh) (8). Moreover, as expected, piscine Sertoli cells express *fshr*, which regulates Sertoli cell proliferation and functioning. Despite this broader range of *fshr* expression and hence bioactivity of Fsh in fish, loss of *fshr* function in zebrafish or medaka was compatible with normal spermatogenesis and fertility, although the start of puberty was delayed in *fshr*^−/−^ zebrafish (10), and *fshr*^−/−^ medaka tended to show a smaller testis (11). This suggests that Lhcgr-mediated sex steroid production eventually may allow testis maturation and spermatogenesis to occur. In this regard, it is not surprising that in zebrafish, clearly disturbed spermatogenesis and loss of fertility required the interruption of both Lhcgr- and Fshr-mediated signaling (10). Yet, in medaka, males remained fertile even after the loss of both, Lhcgr and Fshr function, although testis weight and the number of spermatozoa produced were clearly reduced (11).

However, it is important to note that the role of gonadotropins in regulating spermatogenesis may vary among different teleost fish species in context with the wide range of reproductive strategies found in this largest group of vertebrates. For instance, in several salmonid fishes like Atlantic salmon (12), rainbow trout (13), and various Pacific salmon species (14), which exhibit either annual reproductive cycles or a single reproductive cycle in their lifetime, the onset of puberty and the subsequent rapid testicular growth phase leading to spermatogenesis occur when Lh concentrations in the blood are either undetectable or very low. In contrast, Fsh is readily detectable in the blood of rainbow trout and Pacific salmon species during this process (13, 14). Hence, Lh seems dispensable for the initiation of puberty and for the testicular growth period, until it is eventually secreted close to the spawning season, when spermatogenesis is already complete but when maximum sex steroid levels are observed in context with the differentiation of secondary sexual characters and reproductive behavior. It therefore appears that the initiation of and progression through puberty in male salmonids depends on Fsh signaling. Indeed, previous work has shown that plasma concentrations of Fsh and of 11-ketotestosterone (11-KT), the main androgen in fish (15) jointly increase at the start of salmon puberty (13, 14), when Sertoli cells and type A spermatogonia show elevated levels of proliferation activity (16). In addition, heritable traits (17, 18), body mass/nutrition as well as salinity, photoperiod and water temperature are important parameters regarding male puberty (19-21). Considering the strict relation between Fsh and the initiation of puberty, we hypothesize that conditions modulating the timing of puberty do so by modulating Fsh release/action.

The aim of this study was to examine the hypothesis that puberty in male Atlantic salmon is blocked if the *fshr* gene is nonfunctional, a phenotype different from zebrafish (10) and medaka (11) *fshr*^−/−^ males. Indeed, in contrast to these 2 fish species and also different from observations in mouse mutants (4), we find that loss of *fshr* in Atlantic salmon males leads to failure to enter puberty, confirming a strong Fsh dependency of testis maturation in salmon. Also, the incomplete loss of Fshr function, such as in the mosaic F0 generation or in F1 males with a single amino acid loss in the Fshr protein, caused deviating testis maturation phenotypes. These results will allow developing new avenues to sterility to achieve genetic containment in salmon aquaculture.

## Materials and Methods

### Guide RNA Design and Synthesis

**Figure 1.**
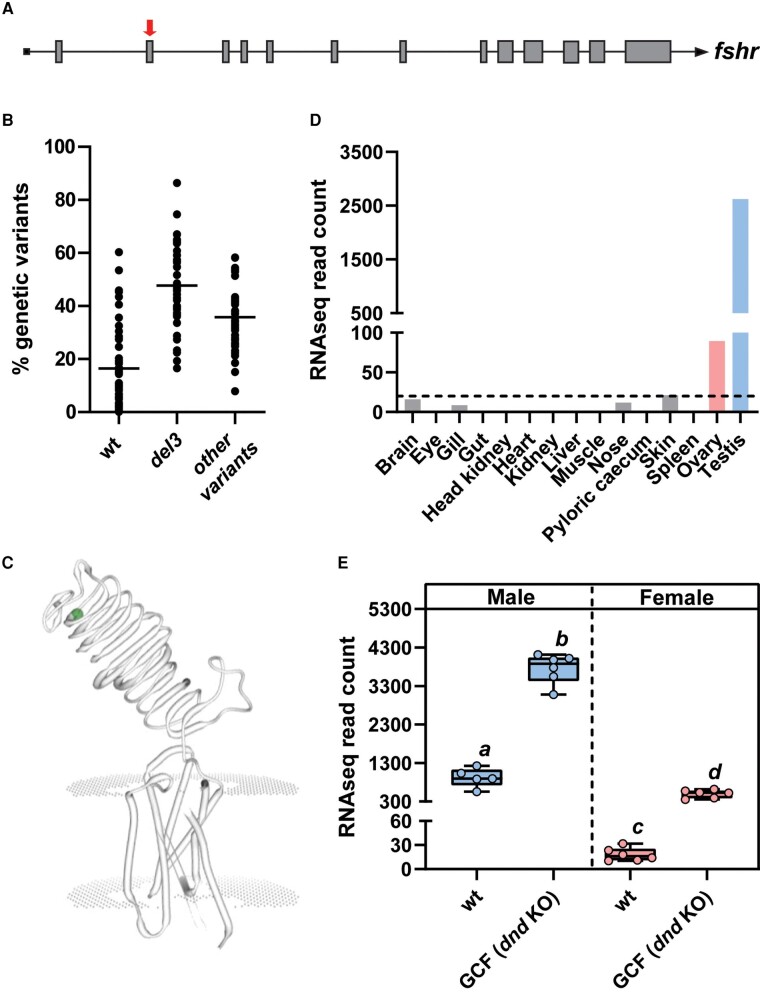
*Fshr* gene exon 2 target site (A; red arrow), The distribution of *fshr* variants in fin clips obtained from 40 *fshr* crispant males (B), dots representing prevalence of variant types found in single fish and the horizontal line the average percentage of each *fshr* variant groups which includes the wt *fshr* variant, deletion 3 (del3) variant and other *fshr* variants (mainly frameshift) deviating from wt *fshr*. In the SWISS-MODEL illustration of the Fshr protein (C), the CRISPR target site is indicated by the green dot. (D) *fshr* expression levels in juvenile tissues used for generating the reference annotation of the Atlantic salmon genome (GenBank GBRB00000000.1). Results are expressed as RNA sequencing (RNA-seq) normalized read counts (n = 1). Dashed line indicates 20 RNAseq reads. (E) Gonadal *fshr* expression in adult wild-type (wt) and germ cell–free (GCF) dead end (*dnd*) knockout salmon males and females. Data are shown as mean RNA-seq normalized read counts ± SEM (n = 5-6; different letters denote statistically significant differences).

High scoring guide RNA (gRNA) target sequences were predicted using an online tool as previously described (22). Templates for producing gRNAs were then made according to protocols published by Gagnon and coworkers (23). The *fshr* guide, 5′-GGAGGTTGGTGAAGGCTTCT-3′, was designed to target exon 2 in *fshr* (Gene ID: 100135779, Fig. 1A) encoding the leucine-rich repeat located on the N-terminal side in the extracellular part of the receptor (see Fig. 1C). The structure of salmon Fshr was modeled by homology modeling to the closest resembling structure using SWISS-MODEL (https://swissmodel.expasy.org). Cas9 mRNA was prepared as previously described (24).

### Microinjection and Embryo Rearing

Fish rearing took place at the Institute of Marine Research, Matre Research Station, Norway. Microinjection of about 2000 freshly fertilized Aquagen strain eggs was done in November 2015, as previously described (24). Two gRNAs were used, targeting *fshr* and *slc45a2*. The gRNAs were coinjected with mRNA encoding Cas9. We targeted *slc45a2* in addition to *fshr,* as previous work has shown that the phenotype of the loss of *slc45a2*, loss of pigmentation, is a reliable indicator of the efficiency of a coinjected, second gRNA (25). All embryos were kept at 8 °C until start fed in March 2016. Full or partial albinos as well as pigmented noninjected control alevins were reared under standard conditions until sorting in September 2016 (see below).

### *fshr* Crispant F0 Rearing

At 10 months of age (September 2016) all fish were placed in a common garden setup; *fshr* crispants displaying partial or full loss of pigmentation (n = 137) together with an equal number of noninjected control (pigmented) fish. At 16 months of age, all fish were fin clipped and passive integrated transponder tagged for identification. Genomic DNA (gDNA) was extracted from the sampled fin tissue using the DNAdvance Genomic DNA Isolation kit (Beckman Coulter, Inc.) according to the manufacturer’s protocol. We also identified the genetic sex by assaying the *sdy* alleles on gDNA according to Ayllon et al (26) but with the following corrected primers and probe: Ss_sdY_Exon2_F: 5′-CCTACAAGCCCTTCTCCCTGAT-3′; Ss_sdY_Exon2_R: 5′-GGGCTTTGGGAGAGAGATGAC-3′; Ss_sdY_Exon2_Pro: 5′-VIC-ATGGATGGGATCCC-MGBNFQ-3′; Ss_sdY_Exon4_R: 5′- GGAGGACTCAAGCCAGATCCT-3′.

### *fshr* Crispant F0 Males, Postsmolt Maturation Regimen and Samplings

On January 25, 2017, a postsmolt maturation regimen started in freshwater, by exposing 14-month-old fish to 16 °C and continuous light for 6 weeks to stimulate early male maturation (27). Fish were then transferred to brackish (25 ppm) water and reared at ambient temperature onwards. On January 24, February 21, May 9, and September 26, 2017, 10 to 20 *fshr* crispants and 10 to 20 wild-type (wt) controls were sampled (Table S1 (28)). The fish were anesthetized with 2 mL/L Finquel vet, measured for weight and length, and blood samples were collected. The fish were sacrificed by cutting into the medulla oblongata, and pituitary and gonads were excised. The pituitary was immersed in RNAlater (Thermo Fisher Scientific) for subsequent RNA extraction. The gonads were weighed, and the gonadosomatic index (GSI) was calculated as GSI = gonad weight (g) × 100/total body weight (g). A small fragment of each gonad was fixed in 4% glutaraldehyde for plastic embedding (Technovit 7100; Kulzer) and histological analyses, as previously described (25, 29). In brief, we examined qualitatively the appearance of the intratubular Sertoli cells and the interstitial Leydig cells and we examined the stage of spermatogenesis by recording the most developed germ cell type. Finally, the incidence of germ cell apoptosis was noted as a relative score (low, intermediate, high).

### Crossing of F0 *fshr* Crispants

F0 *fshr* crispants of both sexes were reared in brackish water (25 ppm) during the winter 2017/2018 and transferred to freshwater in June 2018. In September 2018, all visually immature fish were terminated, and gonad size and maturity status were noted. In November 2018, we also terminated the mature F0 *fshr* crispants, except for the fish kept for breeding purposes (see below).

Since female *fshr* crispants were not sampled during ovarian maturation, we have no information on the possible effects of *fshr* mutagenesis on the female reproductive phenotype. However, in November 2018, 1 year after most male crispants had matured, 48% of the *fshr* crispant females (n = 61) and 63% of the wt females (n = 24) had ovulated and could be stripped for egg collection.

Five highly mutated *fshr* crispant broodstock fish (see Table S3 (28)), identified by sequencing as described below, 2 males and 3 females, were chosen for crossings to produce an F1 generation in November to December 2018. All males were crossed against all females, resulting in 6 crosses. We did not measure larval survival rate in crosses, however there were normal numbers of offspring in all crosses and all larvae were reared in a common garden.

### *fshr* F1 Mutants Rearing, Postsmolt Maturation Stimulation and Sampling

*fshr* F1 alevins hatched in March 2019 and were maintained at 6 °C until start feeding in May 2019, reared in common garden with control (pigmented) fish under standard conditions. All fish were passive integrated transponders tagged and fin clipped as described above for the F0 *fshr* crispants.

On February 4, 2020, F1 mutant and control males were exposed to the same early maturation regimen (16 °C and continuous light for 6 weeks) as the F0 crispants. After termination of the stimulatory regimen fish were reared at ambient conditions until sampling on October 15, 2020, and February 26, 2021 (Table S4 (28)). The samplings were performed in the same manner as the samplings of the F0 *fshr* crispants, described in detail above.

### Mutation Analysis

To assess the rates of mutation types in fin clip DNA of a subset of F0 crispants and F1 mutants, amplicon sequencing was performed similarly to a previously described protocol (23, 30), for sequencing on the MiSeq platform (Illumina). A 2-step polymerase chain reaction (PCR) using Q5 High-Fidelity DNA Polymerase (NEB) was used, first amplifying the *fshr* CRISPR target region (forward primer 5′-tctttccctacacgacgctcttccgatctGCTGTATCGTTCCCAGCAAT-3′ and reverse primer 5′-tggagttcagacgtgtgctcttccgatctCGTTCCTAAGGAGACAAACCA-3′), followed by barcoding with dual-index adapters. A sequencing library was made by pooling equal volumes of all resulting PCR products, followed by gel extraction using QIAquick Gel Extraction Kit (Qiagen) and subsequent DNA quantification on a Qubit fluorometer (Thermo Fisher Scientific). Amplicons were sequenced using MiSeq Kit v. 3 (Illumina) with 300 bp paired-end reads. Processing of sequenced reads was performed as follows. Reads were required to contain the primer sequences in the 5′ ends, with 1 mismatch allowed. Forward and reverse reads were assembled to improve sequence quality for each read-pair. Sequences were mapped to the reference amplicon sequence using Muscle (v. 3.8.1551, (31)) and divided into categories based on presence of indels resulting in frameshift or in-frame mutations, or no indels.

### RNA Extraction and cDNA Preparation

Pituitaries were stored in RNAlater, homogenized in 400 µL of homogenization buffer and processed according to the Maxwell HT-simplyRNA kit instructions (Promega) on a BioMek 4000 instrument (Beckton Dickinson). Quantity and purity of RNA samples were assessed by spectrophotometry on a Nanodrop ND-1000 instrument (Thermo Fisher Scientific). cDNA was prepared by reverse transcription of 200 ng of RNA using the SuperScript IV VILO Master Mix with ezDNase Enzyme, according to the manufacturer's recommendations (Thermo Fisher Scientific).

### Quantitative Real-time PCR

All quantitative PCR (qPCR) assays used in this study were previously published (19, 32, 33). A qPCR reaction was prepared to contain 800 nM of each forward and reverse primer, 250 nM of the probe in a 6-µL reaction containing 1× concentration of the TaqMan Fast Advanced Master Mix (Thermo Fisher Scientific) and 2 µL of a 1/20 diluted cDNA. The reaction was subjected to thermocycling in a QuantStudio 5 Real-Time PCR system (Thermo Fisher Scientific) with an initial hold at 50 °C for 2 minutes followed by an initial denaturation step at 95 °C for 2 minutes. Thermocycling was conducted for 40 cycles using a denaturation step at 95 °C for 1 second followed by a combined annealing and extension step at 60 °C for 30 seconds. Data was processed at Thermo Fisher cloud using the relative quantification app. No-template controls for each gene were run in all qPCR plates. The relative gene expression level was calculated using the comparative Ct (2^−DDCt^) method (34). All values were normalized to *ef1a* and calibrated to the DCt of the immature wt fish.

### 11-KT Quantification by Enzyme-Linked Immunosorbent Assay

11-KT levels were analyzed by enzyme-linked immunosorbent assay (35) in extracted plasma samples from male salmon, as previously described (33). Acetylcholine esterase–labeled tracers and microplates precoated with monoclonal mouse antirabbit IgG were supplied by Cayman Chemicals (Cat# 582751, RRID:AB_2827728). Anti-11-KT was a kind gift from David E. Kime (Sheffield University, UK).

### Statistical Analysis

Statistical analyses were performed using GraphPad Prism 9.4.1 (GraphPad Software, Inc.). All datasets were tested for normal distribution using a D’Agostino & Pearson omnibus normality test. A difference between groups was considered significant when *P* < .05. Unpaired t test was applied where the datasets to be compared passed the normality test. For datasets without normal distribution or with too low n (n < 8) to test for normality, the nonparametric 2-sided Mann–Whitney test was applied. The time wise difference within wt and *fshr* crispant F0 males were analyzed with Kruskal–Wallis test followed by Dunn's multiple comparisons test as was the correlation between genotypes in the F1 generation. Results are presented as means ± SEM.

## Results

### CRISPR Target Region *fshr* Gene and *fshr* Expression in wt Salmon

Our gRNA targeted a region in exon 2, which translates to the N-terminal end of the leucine-rich repeat area of the extracellular part of the Fshr protein (Fig. 1A and 1C). CRISPR-Cas9 caused a mosaic pattern of *fshr* variants in the F0 fish, a mixture of both in-frame and frameshift variants, in addition to wt alleles (see Fig. 1B; Table S2 (28)). Only 16.5 ± 15.8% of detected *fshr* variants in the fin clip of crispants were identical to the wt sequence. The most common *fshr* variant in F0 fin clips was an in-frame deletion of 3 nucleotides (del3), representing 47.7 ± 15.3% of all variants found in the F0 fish. This deletion results in the loss of 1 amino acid in the Fshr protein, glutamine (E) at position aa72 (see Fig. 1C, indicated by a green dot), corresponding to a part of the extracellular domain most distant from Fsh binding grove, and located in the leucine-rich domain closest to the extracellular N-terminal domain of the protein. In general, individual *fshr* crispants displayed a variable mixture of *fshr* variants. The in silico screening of 14 salmon tissue RNA-seq libraries (36) showed that, in juvenile salmon, the expression of *fshr* is only detected at significant levels in ovarian and testicular tissues (Fig. 1D). Previously obtained RNA-seq data from salmon male gonads (37) and unpublished data from the same study from females showed a significant 4-fold and 27-fold increase in *fshr* read counts in germ cell-free testicular and ovarian tissue, respectively, compared to wt control gonads (Fig. 1E). This demonstrates that *fshr* expression primarily originates from somatic cells.

### Testis and Pituitary Phenotypes in *fshr* F0 Crispants

To stimulate testis maturation in postsmolt crispants and controls, fish were exposed to a maturation-inducing regimen for 6 weeks (January 25 to March 15, 2017) (27). Between January 24 and September 26, a total of 64 crispant and 52 wt males were sampled on 4 occasions (see Table S1 (28)); the macroscopic appearance of selected males and their testes is shown in Fig. S1 (28), testis histology in Fig. S2 (28)), and GSI, 11-KT, and pituitary gene expression data are shown in Fig. S3 (28). On January 24 (initial sampling), F0 crispants and wt controls showed similar GSI and plasma 11-KT values (Fig. S3A and B (28)). The testes contained either only type A (Fig. S2A (28)), or type A and type B spermatogonia; the latter were found in half of the controls but in only a quarter of the crispants.

One month (1 m, February) after the start of the maturation regimen, 11-KT levels had increased similarly in crispants and control, while GSI values were significantly lower in crispants than in controls (Fig. S3A and B (28)).

After 4 months, GSI and 11-KT levels had increased further in both groups but crispants showed significantly lower GSI and 11-KT levels than controls and a higher frequency of immature males (4 m, Fig. S3A and B (28)). Histological analysis revealed that the testes of all maturing males sampled in both groups contained all stages of germ cells (Fig. S2C (28)), while apoptotic germ cells appeared to be more frequent in crispants than in control (Fig. S2D (28)).

**Figure 2.**
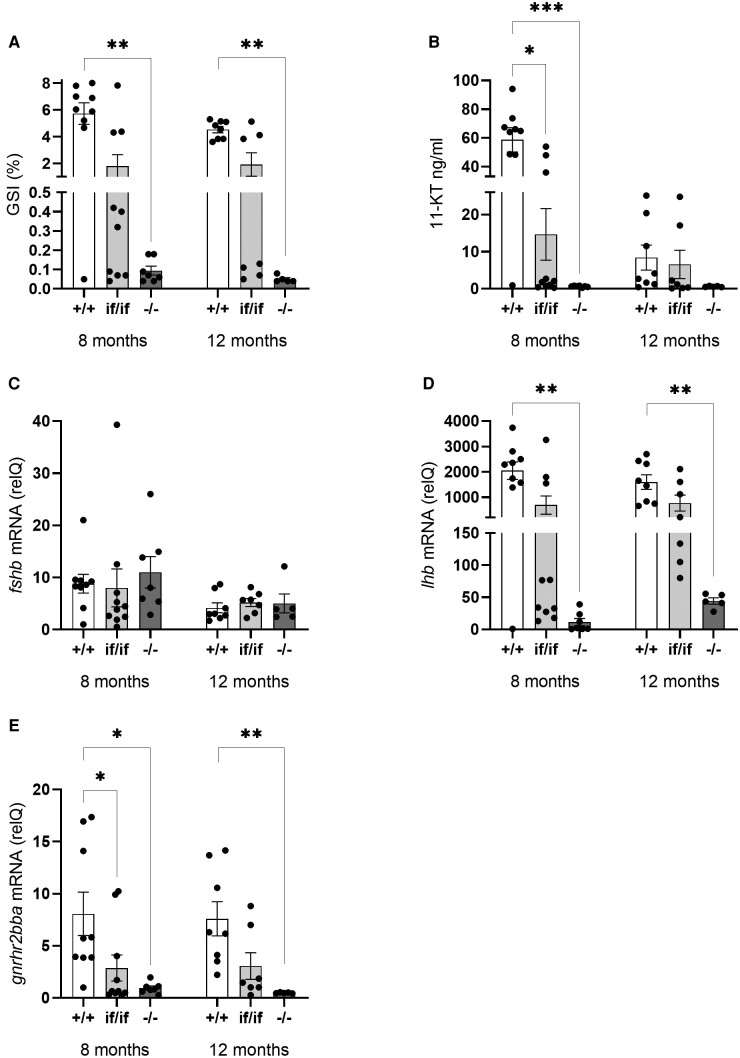
Gonadosomatic indices (GSI; A), plasma 11-ketotestosterone (11-KT; B), pituitary *fshb* (C), *lhb* (D), and *gnrhr2bba* (E) mRNA levels in F1 salmon males, showing *fshr* wild-type (+/+), double allelic in-frame (if/if), or double allelic loss of function mutation (−/−) genotypes, sampled at 8 and 12 months after the start of the maturation-inducing treatment (6 weeks of constant light and 16 °c water temperature, starting February 4, 2020). Data are presented as mean ± SEM. Significant differences between genotypes are indicated by asterisks (*P < .05, **P < .001, ***P < .0001).

At 8 months most controls had matured further and mean plasma 11-KT levels had tripled (8 m, Fig. 2A; Fig. S3A and B (28)). GSI values remained stable, and all germ cell stages were still present, but spermatozoa had become the dominating germ cell type in control males (Fig. S2E (28)). This description was also valid for one-third of the crispants (Fig. S3A and S2F (28)). However, histological analysis identified 2 more subgroups among the crispants. One subgroup had progressed even further than the wt controls, such that only a few small groups of developing germ cells remained, and spermatozoa had become the overwhelmingly dominant germ cell type (Fig. S2G (28)), associated with a mean GSI value of 2.2 (Fig. 2A (28)). Another subgroup of crispants showed small spermatogenic tubuli containing type A spermatogonia and residual sperm (ie, were in postspawning condition) (Fig S2H (28)). The remaining 3 crispants had remained immature. Pituitary transcript levels of *fshb*, *lhb*, and *gnrhr2bba* changed during pubertal testis development according to the maturational status (Fig. S3C-E (28)). The differences found between wt controls and F0 crispants mostly reflected the differences also found in GSI levels (Fig. S3A (28)).

### *fshr* Mutation Types in F1 Salmon

The study aimed to investigate male maturation and *fshr* function. To prevent the inclusion of females in the sampling, *sdy* genotyping was employed to distinguish and remove genetic females from the maturation experiment with F1 mutants. The CRISPR target region in the *fshr* gene was sequenced to identify *fshr* genotypes of individual F1 fish (Tables 1 and 2); 12 mutants carried double allelic loss-of-function mutations, *fshr^−/−^*, 17 males displayed double allelic in-frame mutations, *fshr^if/if^* and 2 males were not mutated and showed double allelic wt, *fshr^+/+^*. Also, 15 wt *fshr^+/+^* control males were included in the common garden (see Table S4 (28) for a full overview of samples, including mutation types) (ie, the total number of wt *fshr^+/+^* in the common garden were 17). In addition, 7 males displayed 1 in-frame mutation in combination with either a frameshift mutation or 1 wt allele. Of these males, 6 matured like wt controls while the remaining 2 stayed immature (see Table 1); this heterogenous group will not receive further attention. Instead, we focus on the wt, *fshr^if/if^* and *fshr^−/−^* males.

**Table 1.** Overview of F1 fish sequenced to identify the *fshr* genotypes of individual fish (ID); 12 male mutants carried double allelic loss of function mutations, −/−, 17 males displayed double allelic in-frame mutations, if/if

Sampling month	ID	Maturity	Genotype	Mutation
8	269	Immature	−/−	del 1/del 1
8	271	Immature	−/−	del 8/del 8
8	280	Immature	−/−	del 1/del 1
8	283	Immature	−/−	del 17/del 17
8	287	Immature	−/−	ins 17/ins 17
8	292	Immature	−/−	ins 8/ins 8
8	294	Immature	−/−	del 17/del 17
12	317	immature	−/−	del 17/del 14
12	322	Immature	−/−	del 1/ins 8
12	324	Immature	−/−	4I4M3I
12	342	Immature	−/−	del 17/del 17
12	344	Immature	−/−	del 1/del 1
8	278	Immature	if/if	del 3/del 3
8	279	Mature	if/if	del 3/del 3
8	284	Mature	if/if	del 3/del 3
8	288	Immature	if/if	del 3/del 3
8	290	Immature	if/if	del 3/del 3
8	291	Immature	if/if	del 3/del 3
8	293	Immature	if/if	del 3/del 3
8	296	Immature	if/if	del 3/del 3
8	297	Immature	if/if	del 3/del 3
8	299	Mature	if/if	del 3/del 3
12	310	Immature	if/if	del 3/del 3
12	320	Mature	if/if	del 3/del 3
12	339	Immature	if/if	del 3/del 3
12	340	Mature	if/if	del 3/del 3
12	346	Immature	if/if	del 3/del 3
12	349	Mature	if/if	del 3/del 3
12	350	Immature	if/if	del 3/del 3
8	270	Mature	if/fs	del 3/ins7
8	281	Mature	if/fs	del 3/del 1
8	282	Immature	if/fs	del 3/del 8
12	323	Mature	if/fs	del 3/del 17
12	348	Mature	if/fs	del 3/ins 8
8	273	Mature	if/wt	del3/wt
12	312	Mature	if/wt	del 3/wt
8	272	Immature	+/+	
12	341	Mature	+/+	

In addition, 7 males displayed 1 in-frame mutation in combination with either a frameshift mutation (if/fs) or 1 wt allele (if/wt) and 2 fish were not mutated (+/+).

**Table 2.** *fshr* genotype and resulting sequence of exon 2 in the individuals (ID) used in this study

ID	Genotype	Sequences (Exon 2; 5′-3′)
	Wt	GGAGTTCAAACAGACGCACATCAGAGTGTTTCCCCG**AGAAGCCTTCACCAACCTCC**TGCAGCTCACTGCCAT
269	−/−	GGAGTTCAAACAGACGCACATCAGAGTGTTTCCCCGAGAAA-CTTCACCAACCTCCTGCAGCTCACTGCCAT
271	−/−	GGAGTTCAAACAGACGCACATCAGAGTGTTTCC--------CCTTCACCAACCTCCTGCAGCTCACTGCCAT
280	−/−	GGAGTTCAAACAGACGCACATCAGAGTGTTTCCCCGAG-AGCCTTCACCAACCTCCTGCAGCTCACTGCCAT
		GGAGTTCAAACAGACGCACATCAGAGTGTTTCCCCGAGAA**A**-CTTCACCAACCTCCTGCAGCTCACTGCCAT
283	−/−	GGAGTTCAAACAGACGCACATCAGAGTGTTTCCC**TT**A-----------------C**A**TGCAGCTCACTGCCAT
287	−/−	GGAGTTCAAACAGACGCACATCAGAGTGTTTCCCCGA**TGT**GCCT**C**C**TG**CA**GAGTGTTTCCCCGATGTG**CCTCCTGCAGCTCACTGCCAT
292	−/−	GAGTTCAAACAGACGCACATCAGAGTGTTTCCCCGAGA**G**G**GGAAACGCA**CTTCACCAACCTCCTGCAGCTCACTGCCAT
294	−/−	GGAGTTCAAACAGACGCACATCAGAGTGTTT-----------------CAACCTCCTGCAGCTCACTGCCAT
317	−/−	GGAGTTCAAACAGACGCACATCAGAGTGTTT-----------------CAACCTCCTGCAGCTCACTGCCAT
		GGAGTTCAAACAGACGCACATCAGAGTGT--------------TTCACCAACCTCCTGCAGCTCACTGCCAT
322	−/−	GGAGTTCAAACAGACGCACATCAGAGTGTTTCCCCGAG-AGCCTTCACCAACCTCCTGCAGCTCACTGCCAT
		GGAGTTCAAACAGACGCACATCAGAGTGTTTCCCCGAGA**G**G**GGAAACGCA**CTTCACCAACCTCCTGCAGCTCACTGCCAT
324	−/−	GGAGTTCAAACAGACGCACATCAGAGTGTTTCCCC**ACATC**AGA**GTGTTT**CTTCACCAACCTCCTGCAGCTCACTGCCAT
342	−/−	GGAGTTCAAACAGACGCACATCAGAGTGTTT-----------------CAACCTCCTGCAGCTCACTGCCAT
344	−/−	GGAGTTCAAACAGACGCACATCAGAGTGTTTCCCCGAGAA**A**-CTTCACCAACCTCCTGCAGCTCACTGCCAT
278	if/if	GGAGTTCAAACAGACGCACATCAGAGTGTTTCCCCG---AGCCTTCACCAACCTCCTGCAGCTCACTGCCAT
279	if/if	GGAGTTCAAACAGACGCACATCAGAGTGTTTCCCCG---AGCCTTCACCAACCTCCTGCAGCTCACTGCCAT
284	if/if	GGAGTTCAAACAGACGCACATCAGAGTGTTTCCCCG---AGCCTTCACCAACCTCCTGCAGCTCACTGCCAT
288	if/if	GGAGTTCAAACAGACGCACATCAGAGTGTTTCCCCG---AGCCTTCACCAACCTCCTGCAGCTCACTGCCAT
290	if/if	GGAGTTCAAACAGACGCACATCAGAGTGTTTCCCCG---AGCCTTCACCAACCTCCTGCAGCTCACTGCCAT
291	if/if	GGAGTTCAAACAGACGCACATCAGAGTGTTTCCCCG---AGCCTTCACCAACCTCCTGCAGCTCACTGCCAT
293	if/if	GGAGTTCAAACAGACGCACATCAGAGTGTTTCCCCG---AGCCTTCACCAACCTCCTGCAGCTCACTGCCAT
296	if/if	GGAGTTCAAACAGACGCACATCAGAGTGTTTCCCCG---AGCCTTCACCAACCTCCTGCAGCTCACTGCCAT
297	if/if	GGAGTTCAAACAGACGCACATCAGAGTGTTTCCCCG---AGCCTTCACCAACCTCCTGCAGCTCACTGCCAT
299	if/if	GGAGTTCAAACAGACGCACATCAGAGTGTTTCCCCG---AGCCTTCACCAACCTCCTGCAGCTCACTGCCAT
310	if/if	GGAGTTCAAACAGACGCACATCAGAGTGTTTCCCCG---AGCCTTCACCAACCTCCTGCAGCTCACTGCCAT
320	if/if	GGAGTTCAAACAGACGCACATCAGAGTGTTTCCCCG---AGCCTTCACCAACCTCCTGCAGCTCACTGCCAT
339	if/if	GGAGTTCAAACAGACGCACATCAGAGTGTTTCCCCG---AGCCTTCACCAACCTCCTGCAGCTCACTGCCAT
340	if/if	GGAGTTCAAACAGACGCACATCAGAGTGTTTCCCCG---AGCCTTCACCAACCTCCTGCAGCTCACTGCCAT
346	if/if	GGAGTTCAAACAGACGCACATCAGAGTGTTTCCCCG---AGCCTTCACCAACCTCCTGCAGCTCACTGCCAT
349	if/if	GGAGTTCAAACAGACGCACATCAGAGTGTTTCCCCG---AGCCTTCACCAACCTCCTGCAGCTCACTGCCAT
350	if/if	GGAGTTCAAACAGACGCACATCAGAGTGTTTCCCCG---AGCCTTCACCAACCTCCTGCAGCTCACTGCCAT
270	if/fs	GGAGTTCAAACAGACGCACATCAGAGTGTTTCCCCG---AGCCTTCACCAACCTCCTGCAGCTCACTGCCAT
		GGAGTTCAAACAGACGCACATCAGAGTGTTTCCCC**ACATC**AGA**GTGTTT**CTTCACCAACCTCCTGCAGCTCACTGCCAT
281	if/fs	GGAGTTCAAACAGACGCACATCAGAGTGTTTCCCCG---AGCCTTCACCAACCTCCTGCAGCTCACTGCCAT
		GGAGTTCAAACAGACGCACATCAGAGTGTTTCCCCGAG-AGCCTTCACCAACCTCCTGCAGCTCACTGCCAT
282	if/fs	GGAGTTCAAACAGACGCACATCAGAGTGTTTCCCCG---AGCCTTCACCAACCTCCTGCAGCTCACTGCCAT
		GGAGTTCAAACAGACGCACATCAGAGTGTTTCC--------CCTTCACCAACCTCCTGCAGCTCACTGCCAT
323	if/fs	GGAGTTCAAACAGACGCACATCAGAGTGTTTCCCCG---AGCCTTCACCAACCTCCTGCAGCTCACTGCCAT
		GGAGTTCAAACAGACGCACATCAGAGTGTTTCCC**TT**A----------------C**A**TGCAGCTCACTGCCAT
348	if/fs	GGAGTTCAAACAGACGCACATCAGAGTGTTTCCCCG---AGCCTTCACCAACCTCCTGCAGCTCACTGCCAT
		GGAGTTCAAACAGACGCACATCAGAGTGTTTCCCCGAGA**G**G**GGAAACGCA**CTTCACCAACCTCCTGCAGCTCACTGCCAT
273	if/wt	GGAGTTCAAACAGACGCACATCAGAGTGTTTCCCCG---AGCCTTCACCAACCTCCTGCAGCTCACTGCCAT
		GGAGTTCAAACAGACGCACATCAGAGTGTTTCCCCGAGAA**A**CCTTCACCAACCTCCTGCAGCTCACTGCCAT
312	if/wt	GGAGTTCAAACAGACGCACATCAGAGTGTTTCCCCG---AGCCTTCACCAACCTCCTGCAGCTCACTGCCAT
		GGAGTTCAAACAGACGCACATCAGAGTGTTTCCCCGAGAAGCCTTCACCAACCTCCTGCAGCTCACTGCCAT

Deletions are indicated with dash (−), substitutions in bold, and insertions in bold and underline. The top row displays the *fshr* wt sequence with the CRISPR target sequence indicated in bold.

### Testis and Pituitary Phenotypes in *fshr* F1 Mutants

**Figure 3.**
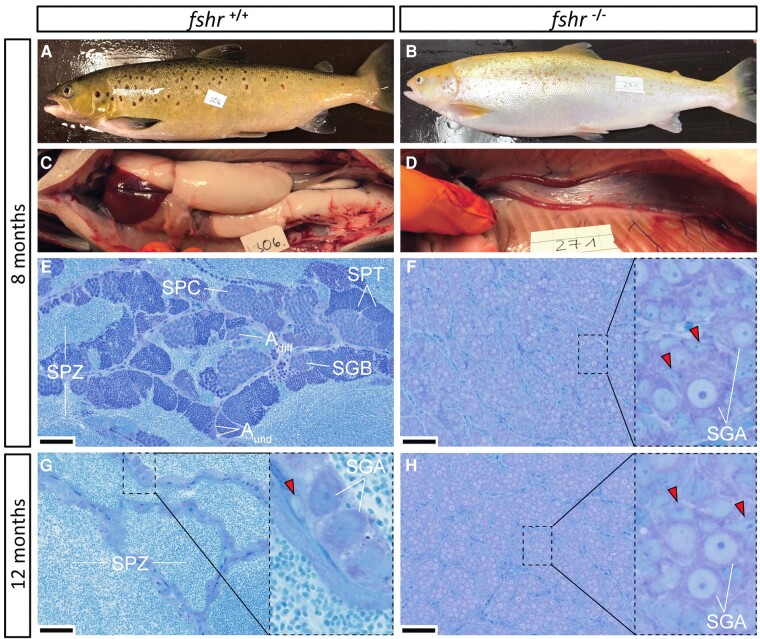
External appearance, macroscopic testis anatomy and histological sections of testes from mature wild-type control (*fshr*^+/+^) and F1 *fshr*^−/−^ males sampled 8 months (A-F) and histological sections from testes sampled 12 months (G-H) after the start of the maturation-inducing treatment (6 weeks of constant light and 16 °C water temperature, starting February 4, 2020). At 8 months all but 1 control males showed the typical external appearance of maturing fish (A) and exhibited large testes upon dissection (C), containing all stages of spermatogenesis (E), including many spermatozoa in the tubular lumen. At 12 months, only 2 types of germ cells remained in the tubuli of control males: a large number of spermatozoa in the lumina and type A spermatogonia (SGA nuclei indicated) enveloped by 1 or 2 Sertoli cells (red arrowheads denote Sertoli cell nuclei) resided on the tubular wall (G). All F1 *fshr*^−/−^ males showed an immature external appearance (B) and exhibited small testes (D) containing only type A spermatogonia (F, H). Bars indicate 50 µm. A_und_, type A undifferentiated spermatogonia; A_diff_, type A differentiating spermatogonia; SGB, type B spermatogonia; SPC, spermatocytes; SPT, spermatids; SPZ, spermatozoa.

Based on our hypothesis that loss of Fshr function would block salmon testis maturation, we chose to sample males around spawning, to increase the chance to identify a contrast between animals which had entered puberty and continued maturation or not (Figs. 2 and 3).

All the control males, except 1, matured and had average GSI values of approximately 6% and 4% at 8 and 12 months after the start of the maturation inducing period, respectively (Fig. 2A). All *fshr^−/^*^−^ F1 mutants, on the other hand, showed mean GSI values below 0.1% at both 8 and 12 months (ie, ∼40- to 60-fold lower than the maturing wt controls). At 8 months, histological analysis showed that spermatogenic tubuli of maturing wt controls contained large numbers of spermatozoa in the lumen, while the tubular walls were covered by developing spermatogenic cysts, containing all types of germ cells but mainly spermatocytes and spermatids (Fig. 3E). In stark contrast, the much smaller testes of *fshr^−/^*^−^ F1 mutants showed small, solid spermatogenic tubuli missing a lumen and containing type A spermatogonia as the furthest developed germ cell type (ie, these males were completely immature) (Fig. 3F). At 12 months, the tubuli of wt controls contained only 2 types of germ cells: a large number of spermatozoa filled the lumina, while quiescent type A spermatogonia, surrounded by 1 or 2 Sertoli cells, were located along the tubular wall (Fig. 3G). In contrast, the testis histology of *fshr^−/−^* F1 mutants showed no changes between 8 and 12 months (Fig. 3H), indicating that all males remained immature.

The males showing double allelic in-frame mutations (*fshr^if/if^*) formed 3 subgroups at 8 months: 4 of 10 males remained immature, showing low GSI values and only type A spermatogonia, 3 of 10 had started maturation recently and showed testes with type A and type B spermatogonia and intermediate GSI values, while the remaining 3 showed high GSI values and full spermatogenic activity, similar to maturing wt controls. At 12 months, 2 subgroups remained: 4 of 7 males were immature and 3 of 7 were fully mature, both subgroups showing the corresponding testis histology and GSI values.

Plasma 11-KT levels reflected the state of maturation (Fig. 2B). All *fshr^−/^*^−^ males and the 8 immature *fshr^if/if^* mutants displayed low levels of 11-KT, while high 11-KT levels were recorded at 8 months for maturing *fshr^if/if^* mutants and maturing wt controls. The 3 *fshr^if/if^* mutants that had just started maturation showed a mean plasma 11-KT level (2.2 ng/mL) 4-fold higher than their 4 immature counterparts (0.5 ng/mL); we did not analyze statistical differences in these small subgroups. Mean 11-KT plasma levels decreased ∼6-fold in maturing males at 12 months, irrespective of the genotype, indicating that these males were approaching the end of the spawning season.

The pituitary levels of *fshb* (Fig. 2C) did not differ between the genotypes at either time point but decreased 2-fold from 8 months to 12 months. The levels of *lhb* and *gnrhr2bba* (Fig. 2D and 2E), however, again reflected the maturational pattern seen in the GSI and androgen levels, with low levels in *fshr^−/−^* males and the 8 immature *fshr^if/if^* mutants, while maturing *fshr^if/if^* mutants and wt controls displayed high levels at 8 months. Different from GSI and plasma androgen levels among mature fish, both *lhb* and *gnrhr2bba* remained high also at 12 months.

## Discussion

### Mutation Rate and Type of Mutations Found in F0 and F1

The gRNA guide used to target salmon *fshr* was effective in creating mutated variants of the *fshr* gene, where around 85% of the sequenced *fshr* variants were diverging from the wt sequence in F0 crispant fin clips (Fig. S3, Table S2 (28)). Some *fshr* variants were more prevalent than others, a common feature with targeted mutagenesis using CRISPR-Cas9 in salmon (24, 30, 38). The most common *fshr* variant was an in-frame del3 mutation, which resulted in the loss of 1 amino acid in the encoded protein, a glutamine (E) at position aa72. This glutamine is part of the extracellular domain most distant from the Fsh binding grove. In mammals many phenotype-inducing *FSHR* mutations are known, causing amino acid aberrations in the encoded protein. Amino acid aberrations in this area of the protein, may affect affinity and specificity of FSH binding to its receptor (39). Therefore, we assume that loss of aa72 in salmon Fshr partially impairs aspects of receptor function, potentially rendering Fsh binding and/or receptor activation less efficient. This may explain why half of the *fshr^if/if^* mutant males remained immature, in contrast to only 1 of the 17 wt males, and also why an intermediate phenotype was observed in the subset of the *fshr^if/if^* mutants sampled at 8 months showing a delayed start of spermatogenesis. The absence of such an intermediate group after 12 months may indicate that the males with a delayed start were able to complete maturation during the 4 months period bridging the 2 samplings. Hence, maturation, including testis growth, plasma 11-KT levels and pituitary gene expression is possible for *fshr^if/if^* mutant males, either with a timing like wt controls, or after a delayed start, demonstrating that the receptor is partially active. Given that *fshr^if/if^* mutants can follow different developmental trajectories (no, delayed, or normal maturation), other factors than the mutation itself influenced the decision regarding the specific developmental path taken. In future investigations, we will explore whether differences in Fsh plasma levels play a role in this context. Specifically, we will examine whether elevated circulating Fsh levels can potentially offset the partial functional impairment of the mutated receptor, thus facilitating testis maturation despite the mutation.

F0 crispants also showed a mixture of (1) more immature males than found among wt controls, (2) a delayed start of maturation, or (3) normal maturation. These similarities might be attributed to the prevalence of the del3 mutation and the presence of wt alleles in the F0 crispants. However, unlike the homogenous *fshr^if/if^* mutants with both alleles mutated, the mosaic F0 crispants exhibited an additional deviation from the wt controls. A subset of the maturing crispants displayed a lower GSI at 1, 4, and 8 months than the wt controls. This indicates that although they embarked on maturation, they did so by producing a reduced number of spermatogenic cysts progressing towards maturation compared with the controls. Moreover, at the sampling at 8 months, more than half of the crispants exhibited 2 distinct maturation statuses that were not observed in the wt males. Firstly, a group of 7 males had already completed spermatogenesis, progressing at a faster rate than both normally maturing crispants and wt controls. Secondly, another group of 7 males had advanced even further and entered the regression phase after the spawning season (see Fig. S2H (28)). During this phase, residual sperm is eliminated through phagocytosis by Sertoli cells, usually as preparation for a subsequent reproductive cycle. We propose that the specific observations among the F0 crispants can be explained by an earlier cessation in the production of spermatogenic cysts. These cysts subsequently completed their development but in numbers that fell short of achieving the testis weight/GSI seen in normally maturing males. Collectively, these deviations observed among the F0 crispants suggest that the biological activity of Fsh was partially compromised in terms of an important aspect of Fsh bioactivity: stimulating the production of spermatogenic cysts (40, 41) and, consequently, the number of germ cells generated during a reproductive season.

### Loss of *fshr* in Salmon Compared with Mammalian and Teleost Counterparts

In this study, all *fshr^−/−^* salmon males examined remained in an immature state. This phenotype contrasts with the observed phenotypes in mice, where *Fshr^−/−^* males exhibited reduced testis size due to a decreased number of Sertoli cells (4). However, these mice were still able to produce sperm and sire offspring, albeit with a higher proportion of misshaped sperm in the ejaculate and reduced sperm motility parameters. Interestingly, recent research using new genetic models has demonstrated that a constitutively active FSH receptor in mouse, can stimulate nearly normal spermatogenesis even in the absence of androgen signaling (7). This unexpected finding appears to be partially attributed to the expression of genes typically regulated by androgens but controlled by alternative pathways responding to strong FSH receptor signaling. Thus, although not strictly necessary for spermatogenesis, FSH alone can enhance male fertility in mice. On the other hand, our findings in male salmon suggest that Fsh receptor mediated signaling is crucial for the initiation of spermatogenesis.

No similarity was found between the *fshr^−/−^* male salmon phenotype and 2 other teleost counterparts, namely medaka and zebrafish *fshr^−/−^* males. In these 2 species, the *fshr^−/−^* males still underwent maturation and produced sperm, although zebrafish exhibited a delay in the onset of spermatogenesis and medaka displayed smaller testes (10, 42). In salmonids, the initiation of puberty and subsequent testicular growth phase leading to the completion of spermatogenesis occur when Lh plasma levels are either undetectable or very low, while Fsh is readily detected in the bloodstream of rainbow trout (13) and Pacific salmon species (14). Therefore, in salmon, Lh appears to be dispensable for the onset of puberty and the testicular growth phase when Fsh is the sole circulating gonadotropin present. Also in zebrafish, puberty and testicular growth occurred in the absence of Lh (43). In Atlantic salmon, a rise in Lh plasma levels typically occurs near the spawning when the seasonal gonadal growth is completed (12). This increase aligns with the peak levels of sex steroids, likely linked to the development of secondary sexual characteristics and the display of reproductive behaviors. Hence, it seems that the initiation and progression of puberty in male salmonids, under normal physiological conditions, rely solely on Fsh signaling. This is now supported by the functional evidence presented in this study, as the loss of Fsh receptor function in Atlantic salmon resulted in failure to enter puberty, which sharply contrasts with other vertebrate models, including zebrafish and medaka. Fsh in medaka is a quite potent ligand for the Lh receptor as well (44), so that Fsh may lead to an additional activation of the Lh receptor, potentially explaining the mild phenotype in *fshr*^−/−^ medaka, since Leydig cell products may still trigger spermatogenesis, resulting in fertility (42). Alternatively, it is possible that medaka spermatogenesis has become partially independent of gonadotropin regulation. This assumption arises from the fact that even after the loss of function of both gonadotropin receptors, medaka males still are fertile, albeit with smaller testes and reduced sperm production (11). Additionally, a recent report suggests that androgens/androgen receptors have lost their function in spermatogenesis in this species (45), further supporting the notion of at least partial gonadotropin-independent regulation of medaka spermatogenesis. In zebrafish (10), similar to salmon (46), Fsh is unable to bind to the Lh receptor. Therefore, it is unlikely that Fsh-mediated cross-activation of the Lh receptor contributes to stimulating puberty in zebrafish. In contrast to medaka, the double knockout of the *fshr* and *lhcgr* genes in zebrafish resulted in disrupted spermatogenesis and infertility (10). Thus, it appears that other, yet unidentified mechanisms partially compensate for the loss of FSHR/Fshr, such that mice, medaka, and zebrafish lacking the *Fshr*/*fshr* gene can still undergo maturation and produce sperm. In the case of salmon, these presumed alternative mechanisms were not activated during the examined period. This is evidenced by the fact that all *fshr^−/−^* male salmon remained immature, and histological analysis revealed only the presence of type A spermatogonia up to the age of 2 years and 8 months.

Certain environmental conditions (here, 16 °C and continuous light for 6 weeks) triggered spermatogenic activity, elevated GSI and plasma androgen levels, and changes in pituitary gene expression in wt controls and some of the mutation combinations, including several individuals showing the common in-frame mutation del3. These changes are indicators of having entered puberty as described earlier for Atlantic salmon (16, 19, 27). However, none of these changes were observed in *fshr^−/−^* male salmon sampled at 8 or 12 months after the onset of the puberty stimulation regimen, and such changes were also absent even after 2 years and 8 months. Hence, by not sampling *fshr* F1 mutants shortly after starting exposure to stimulatory conditions we may have missed a potential immediate response. However, we can infer from the absence of any sign of maturation among the *fshr^−/−^* males sampled that none of them has completed pubertal development. From this, we can assume that Fsh is necessary for activating Leydig cell steroidogenesis, likely involving the expression of Fsh receptor in Leydig cells, as demonstrated in Japanese eel (47), African catfish (48), zebrafish (8), and Senegalese sole (9). This is further supported by the Fsh-stimulated androgen secretion observed in various fish species, including salmonids (49). Androgens are known to stimulate spermatogenesis in most fish, including Atlantic salmon (19), so the failure to upregulate androgen production likely contributes to the *fshr^−/−^* phenotype in salmon. Moreover, other aspects of Fsh bioactivity stimulate spermatogenesis independently of androgens. These pathways involve signaling molecules, ranging from growth factors, such as antimullerian hormone, Amh (50), and gonadal soma–derived growth factor, Gsdf (51), to small, lipophilic nonsteroidal molecules like retinoic acid (52) and prostaglandins (53). These sex steroid–independent pathways are also assumed to remain idle in the absence of Fsh signaling. In fish, both interstitial Leydig cells and intratubular Sertoli cells express the Fsh receptor. Further research will determine if all aspects of the phenotype observed in male salmon are a result of the loss of *fshr* and its function in the different testicular Fsh target cell types at the onset of puberty. For instance, loss of Fshr signaling may lead to developmental issues, preventing Leydig and/or Sertoli cells from reaching their full functional competence, rendering these somatic cells unable to support germ cells beyond the stage of type A spermatogonia.

Analysis of pituitary gene expression in F1 *fshr^−/−^* males provided remarkable results. Loss of Fshr signaling was associated with low *lhb* and *gnrhr2bba* transcript levels, suggesting that the maturation-associated increases of these 2 transcripts depend on the Fshr-mediated release of testicular signaling molecules. Previous work has shown that aromatizable androgens like testosterone (T) or estrogens strongly stimulate *lhb* gene expression in salmon (54). More recently, we have shown that not only estrogen but also the nonaromatizable 11-KT increased *lhb* and *gnrhr2bba* transcript levels in Atlantic salmon (55). Therefore, we propose that in *fshr^−/−^* salmon, the Fsh-stimulated production of 11-KT and T, acting as androgens, along with T after conversion to estrogen, is impaired. This deficiency leads to reduced levels of *lhb* and *gnrhr2bba*. Moreover, the absence of elevated plasma levels of 11-KT in *fshr^−/−^* salmon may explain why *fshb* levels in their pituitaries increased to levels found in maturing wt pituitaries. This observation may reflect the direct inhibitory effect of 11-KT, but not of T or estrogens, on *fshb* levels in salmon pituitary tissue culture experiments (55). However, in male germ cell-free (*dead end* knockout), *dnd^−/^*^−^ salmon, where plasma androgens also do not increase during natural puberty, pituitary *fshb* transcript levels remain low at prepubertal levels. An important difference between *fshr^−/−^* and *dnd^−/−^* males is that the latter have lost all germ cells (29, 56). It appears, therefore, that it is not only the low androgen production, but the combination of low androgen levels and the presence of immature germ cells, resulting in a signal that stimulates increased *fshb* levels. Future work will have to clarify the underlying mechanism, potentially relevant for triggering male puberty in salmon.

As mentioned in the Introduction, Fsh and 11-KT plasma levels jointly increase at the start of salmon puberty together with pituitary *fshb* mRNA levels. At the same time, Sertoli cells and type A spermatogonia showed increased proliferation activity. There is a close relation between upregulating pituitary Fsh production and release and the initiation of puberty in male salmon, making it reasonable to assume that loss of a functional Fshr in salmon may block maturation for good. However, long-term studies are still needed to examine the maturational status of *fshr^−/−^* males older than 2 years and 8 months. This will also allow examining maturation in female salmon *fshr*^−/−^ mutants. After all, ovarian maturation is affected more severely by the loss of FSHR/Fshr function in all vertebrates studied so far (4, 10, 11).

Precocious male puberty with associated welfare and growth problems is a common issue in salmon farming (1). Our present findings open a window of opportunity for breeders to produce males by precision breeding, in which mutated fish either do not enter puberty (*fshr*^−/−^) or do so with a lower prevalence (*fshr^if/if^*) which could be used to prevent/reduce maturity in farms, in particular in land-based RAS. Since *fshr*^−/−^ males do not seem to enter puberty, their immature *fshr*^−/−^ germinal stem cells can be transplanted into sterile hosts carrying Fshr on their gonadal somatic cells. These fish are likely to mature and to produce large numbers of *fshr*^−/−^ fish lacking both the ability to enter puberty and to breed with wild fish upon escape. Similar experiments in medaka *fshr^−/−^* mutants have shown this to be a feasible solution to obtain both sterility and prevention of unwanted puberty phenotypes (57). Fish with an *fshr*^−/−^ background may therefore be particularly useful to prevent maturation in land-based RAS production systems for Atlantic salmon.

## Conclusions

We observed that *fshr*^−/−^ male Atlantic salmon stay immature while almost all control fish enter maturity. This is in stark contrast to findings in both medaka and zebrafish where *fshr*^−/−^ males mature and are fertile. The difference may be attributable to the fact that in salmonids initiation of puberty relies on Fsh signaling while in medaka and zebrafish, alternative mechanisms can replace Fshr signaling to achieve sexual maturity. The higher prevalence of immature phenotypes found in *fshr^if/if^* and lack of maturation in male *fshr*^−/−^ Atlantic salmon, may also represent new avenues for puberty prevention and sterility in salmon aquaculture.

## Data Availability

All datasets generated during and/or analyzed during the current study are not publicly available but are available from the corresponding author on reasonable request.

## Nomenclature

The present study follows the ZFIN nomenclature conventions for protein and gene names in mammals and fish (eg, mammalian protein: FSHR; mammalian gene: *Fshr*; fish protein: Fshr; fish gene: *fshr*).

## Abbreviations

CRISPR-Cas9: clustered regularly interspaced short palindromic repeats and CRISPR-associated protein 9
FSH: follicle-stimulating hormone
GnRH: gonadotropin-releasing hormone
GSI: the gonadosomatic index
11-KT: 11-ketotestosterone
LH: luteinizing hormone
LHCGR: luteinizing hormone/choriogonadotropin receptor
qPCR: quantitative real-time polymerase chain reaction
RAS: recirculation aquaculture system
sdY: *s*exually dimorphic on the Y-chromosome
T: testosterone
Wt: wild type
